# Facilitators and barriers to COVID-19 vaccination uptake among ethnic minorities: A qualitative study in primary care

**DOI:** 10.1371/journal.pone.0270504

**Published:** 2022-07-08

**Authors:** Lucia Magee, Felicity Knights, Doug G. J. Mckechnie, Roaa Al-bedaery, Mohammad S. Razai

**Affiliations:** 1 Population Health Research Institute, St George’s, University of London, London, United Kingdom; 2 The Migrant Health Research Group, Institute for Infection and Immunity, St George’s, University of London, London, United Kingdom; 3 Department of Primary Care and Population Health, University College London, London, United Kingdom; University of Leeds, UNITED KINGDOM

## Abstract

**Introduction:**

COVID-19 vaccination effectively reduces severe disease and death from COVID-19. However, both vaccine uptake and intention to vaccinate differ amongst population groups. Vaccine hesitancy is highest amongst specific ethnic minority groups. There is very limited understanding of the barriers and facilitators to COVID-19 vaccine uptake in Black and South Asian ethnicities. Therefore, we aimed to explore COVID-19 vaccination hesitancy in primary care patients from South Asian (Bangladeshi/Pakistani) and Black or Black British/African/Caribbean/Mixed ethnicities.

**Methods:**

Patients from the above ethnicities were recruited using convenience sampling in four London general practices. Telephone interviews were conducted, using an interpreter if necessary, covering questions on the degree of vaccine hesitancy, barriers and potential facilitators, and decision-making. Interviews were transcribed verbatim and thematically analysed. Data collection and analysis occurred concurrently with the iterative development of the topic guide and coding framework. Key themes were conceptualised through discussion with the wider team.

**Results:**

Of thirty-eight interviews, 55% (21) of these were in Black or Black British/African/Caribbean/Mixed ethnicities, 32% (12) in Asian / British Asian and 13% (5) in mixed Black and White ethnicities. Key themes included concerns about the speed of vaccine roll-out and potential impacts on health, mistrust of official information, and exposure to misinformation. In addition, exposure to negative messages linked to vaccination appears to outweigh positive messages received. Facilitators included the opportunity to discuss concerns with a healthcare professional, utilising social influences via communities and highlighting incentives.

**Conclusion:**

COVID-19 has disproportionately impacted ethnic minority groups. Vaccination is an effective strategy for mitigating risk. We have demonstrated factors contributing to vaccine reluctance, hesitancy and refusal and highlighted levers for change.

## Introduction

COVID-19 vaccination is an effective strategy to reduce the spread of infection and prevent severe disease, hospitalisation and death from COVID-19 [1]. More than 11.8 billion doses of COVID-19 vaccines have been administered globally, and 66.8% of the world population has received at least one dose of the vaccine as of May 2022 [2]. In the UK, over 53 million people aged 12 or over have had the first dose, 49.8 million the second dose, 39.5 million the third dose of the COVID-19 vaccine (93%, 87% and 69% of the population, respectively) [3]. Positive attitudes towards the COVID-19 vaccines have increased over time, with 96% of adults reporting positive sentiments in July 2021 compared with 78% in December 2020 [4]. However, evidence suggests that both the vaccine uptake and intention to vaccinate differ amongst population groups [1,4–7]. Vaccine hesitancy, defined by the World Health Organization as a ‘delay in acceptance or refusal of safe vaccines despite availability of vaccine services’ [8], is higher in socio-economically deprived communities (8% in most deprived, 3% in the least deprived areas), younger groups (14%) and some ethnic minorities: in particular, Black and South Asian ethnicities (18% and 17% respectively, compared with 4% in White adults) [9,10]. Disaggregated data by ethnic subgroups show that people identifying as Black Caribbean and Black African have the lowest vaccination rates (66.8% and 71.2%), followed by people of Pakistani backgrounds (78.4% compared with 94% white [11]). The trend of lower uptake of COVID-19 vaccines among ethnic minorities has continued to March 2022, with the proportion of people who received three COVID-19 vaccinations being lower for ethnic minorities (Black Caribbean 38%, Black African 45%, Pakistani 45%, Bangladeshi 55%, Mixed 60%, Indian 73%, White 76%) [12,13].

Vaccine hesitancy is a complex, context-dependent phenomenon [1]. Vaccine hesitant individuals can occupy various positions along the spectrum of vaccine hesitancy perspectives, from refusal and spreading misinformation to acceptance with uncertainty [1]. Vaccine hesitancy rates vary by vaccine type and have been influenced by a range of factors, including geographical location, socio-demographic and cultural factors, social determinants and health inequities [14]. Additionally, recent evidence from the US suggests that lifetime experiences of racial discrimination is associated with 21% increased odds of COVID-19 vaccine hesitancy [15]. In the UK, evidence from 633 ethnic minority adults shows that nearly 1 in 10 people who had refused a COVID-19 vaccine had experienced racial discrimination in a medical setting [16]. The total effect of racial discrimination on refusing the vaccine was nearly fourfold and was mediated by low trust in the healthcare system [16].

Recent quantitative surveys have explored the reasons for COVID-19 vaccine hesitancy, demonstrating concerns about vaccine side effects, long term effects on health, the speed of development of the vaccines and belief in conspiracy theories and misinformation [1,17].

The disparities in COVID-19 vaccine uptake have profound implications. The pandemic has disproportionately impacted ethnic minority groups with much higher infection, hospitalisation, and death rates than White populations [18]. Without an effective mitigation strategy, such as vaccination, the pandemic’s severe and unequal impact will further aggravate health inequalities among these marginalised groups.

There is an urgent need to understand the factors that cause vaccine reluctance, hesitancy, and refusal and how to facilitate engagement with vaccination programmes. Primary care in the UK has had a unique role in vaccine delivery during the pandemic, mobilising to deliver millions of vaccines to the population over a short time frame, particularly to those most vulnerable. This study sought to explore barriers and facilitators to COVID-19 vaccine uptake–in key ethnic groups that have reported higher hesitancy rates–from the unique position of primary care.

## Methods

### Study design

The research team conducted semi-structured qualitative interviews by telephone with patients who identified as belonging to ethnic groups previously identified as most hesitant [1,7,19] and belonging to four GP practices in London. The definition of ethnic categories is based on the UK census groups, which is also how patients are asked to identify their ethnicity on registering with a general practice. Interviews were conducted by one of four team members (MSR, FK, RA, DM) of different ethnicities: two White, one Arab and one Asian and genders (two male and two female) who worked in four different practices. Participants were selected by convenience sampling from these four practices. These practices’ metropolitan areas included inner-city and suburban (but not rural) areas.

### Sampling and recruitment

Participants were identified by a systematic search of the practice electronic systems for all patients aged over 18 who identified as Black British or Black African, Black Caribbean, South Asian (Bangladeshi or Pakistani) ethnicities (or mixed ethnicity pertaining to one of the above), as coded by the practice. Further inclusion criteria included those who could consent, had declined or delayed vaccination, or had not yet received a COVID-19 vaccination. The researchers hand-searched the electronic records for patients who had accepted a COVID-19 vaccination but were significantly delayed compared to their initial invitation. Ethnicity was self-reported. The list of eligible participants was sampled with monitoring of patient demographics throughout the study to recruit a diverse sample regarding age, gender and ethnic grouping. All researchers were practising general practitioners who had decided to receive the COVID-19 vaccines. Potential participants were contacted by the team member working at the practice the patient was registered with and given a brief verbal description of the study by telephone. All individuals interested in participating were emailed, texted or posted a participant information sheet and consent form, and invited to a telephone interview at a mutually convenient time. The consent form and patient information sheet were translated into Bengali, Pakistani Punjabi, and Urdu.

### Ethics and informed consent

Ethics was granted by the Health Research Authority and Care Research Wales (REC 21/2M/0063). Participants were assured that their involvement or refusal to be involved would not affect their clinical care and received no financial reimbursement. All participants who agreed to take part provided written consent and consented to audio recording. The data were analysed anonymously.

### Data collection and analysis

Interviews were carried out between May and August 2021. Although interpreters were offered to all participants in a language of their choice, this offer was taken up in one interview using an interpreter from a professional interpretation service.

Participants were asked to provide demographic data on age, sex, occupation, comorbidities, previous non-COVID vaccine uptake, geographical location, country of birth, and education level. They were asked directly whether they would accept a COVID-19 vaccination if offered. Data collection and analysis occurred concurrently, as common in qualitative research [20]. The authors developed the topic guide collaboratively with reference to existing literature and expert opinion pieces on the topic. They piloted it in two practices on patients of Black and South Asian ethnicities before the commencement of data collection. The topic guide underwent further iterations. Data collection and analysis informed further areas of questioning until thematic data saturation, in which no new themes of interest emerged, was reached [21], as agreed by all authors.

Participants were asked about the COVID-19 vaccines and factors and information sources that had informed their decision-making, including previous experience with healthcare and vaccinations. As the study progressed, further prompts and probes were added to develop a richer understanding of the patient experience and possible themes.

Interviews were audio-recorded, anonymised, transcribed verbatim by a professional transcription service, and checked for accuracy. Transcripts were analysed using NVivo software (version 12). Data analysis was inductive, informed by thematic analysis, as outlined by Braun and Clarke [22]. After the first ten interviews had been transcribed, two researchers (FK and LM) immersed themselves in the data, repeatedly reading the transcripts to develop a collective understanding of participants’ experiences and meaning across the dataset [23]. Key issues, concepts, and themes arising from the data were identified and coded separately and debated between FK, LM and MSR, leading to an initial coding framework that was discussed with the wider research team and developed through negotiated consensus. The framework was refined iteratively by the process of constant comparison by LM. Emerging themes and experiences of interviews were then discussed across the team, feeding into topic guide amendments [24] until the entire team agreed upon a finalised coding framework and theme conception.

## Results

The research team conducted 38 interviews between May and August 2021, with a response rate of 53.5% (38/71). Interviews lasted between 15 and 45 minutes. Of those interviewed, 55% (21) identified as Black/ Black-British, 32% (12) as Asian / British Asian, and 13% (5) as mixed ethnicities. The mean age was 50.5 years, and 58% (22) were female. Around a quarter of participants (26%) had qualifications beyond A-level. Of those who declined to participate (33) in the study, the most common reason given was lack of time. The majority (66%) of those who refused were of Black British ethnicity. Table 1 demonstrates the key characteristics of the participants.

**Table 1:** Demographic and clinical characteristics of the 38 participants interviewed.

**Characteristics**	**Value**
**Age in years**, mean (range)	50.5 (19–82)
**Female**	22 (58%)
**Ethnicity**	
Black/Black British -African	11 (29%)
Black/Black British -Caribbean	10 (26.3%)
Asian/Asian British Pakistani	8 (21%)
Asian/Asian British Bangladeshi	4 (10.5%)
Mixed Caribbean/White	4 (10.5%)
Mixed African/White	1 (2.6%)
**Place of birth**	
UK	19 (50%)
Africa	7 (18.4%)
Caribbean	5 (13%)
South Asia	7 (18.5%)
**Qualifications**	
None	9 (24%)
GCSE/O Levels	5 (13.2%)
A-Levels/BTEC/NVQ	10 (26.3%)
Bachelors	5 (13%)
Masters	5 (13%)
Other	4 (10.5%)
**Comorbidities**	
Hypertension	13 (34%)
Diabetes	7 (18.4%)
Stroke	2 (5.2%)
Depression	2 (5.2%)
Anxiety	4 (10.5%)
None of the above	10 (26.3%)
**Ability to speak English conversationally**	
Yes	37 (97%)
No	1 (3%)
**Any previous immunisation** *	
Yes	32 (84%)
No	6 (16%)
**Willingness to take up COVID-19 vaccine**	
Yes	10 (26%)
No	14 (37%)
Not sure	14 (37%)

*As reported by the patient.

### Degree of COVID-19 vaccine hesitancy

Of those interviewed, 37% participants expressed that they would not get the vaccine, 37% stated that they were not sure yet about getting the vaccine, and 26% said that they intended to get the vaccine or had it already, albeit with a significant delay of weeks or months after the initial invitation. There was a multiplicity of views and concerns raised by participants. Key themes for barriers included concerns about the speed of vaccine rollout, concerns about the vaccines’ known and unknown effects on health, mistrust of official information and exposure to misinformation. Key themes for facilitators included discussing concerns with healthcare professionals, social influences and highlighting incentives.

### Reasons for COVID-19 vaccine hesitancy and mistrust (barriers)

There were multiple reasons given for COVID-19 vaccine hesitancy and mistrust, which reflected the diversity of the participants, and were categorised into key themes as listed below. A practical summary with recommendations to address these barriers is provided in **Table 2.**

**Table 2:** Key Implementation messages: Addressing participants’ views on barriers and facilitators of COVID-19 vaccine uptake.

Factors influencing uptake	Opportunities to address this in primary care / the community	Considerations & Resources for practice:
**Concerns about speed of vaccine roll out**	Addressing fears directly about speed of vaccinxfe roll out	• Explain vaccination research process why this was quicker than anticipated. • Useful links to help guide this process from LSHTM [25] and in the COVID-19 Communication Handbook [26]
**Concerns about the vaccines’ known and unknown effects on health**	Tailored information, addressing how different ethnicities were involved in the study	• Discussing concerns and sharing information with specific information about the demographics of participants in the trials. • There is a short video [27] available from John Hopkins and a Key Questions article [28]
**Information Sources of Participants**	Understand previous misinformation exposure in order to address concerns directly.	• Seek to understand misinformation exposure and how this has informed current thinking and hesitancy around vaccination • The following provide suggestions about how to approach conversations countering conspiracy and misinformation: The Conspiracy Theory Handbook [29] and Tips on Countering Conspiracy & Misinformation [30] • Utilising communication tools [31] to express risks and benefits
**Trust in healthcare professionals**	Opportunity for information provision from trusted Health Care Professionals	• Opportunistic vaccine promotion during health contacts • For those unvaccinated and in at-risk categories, opportunity for discussion with healthcare professional
**Social Influence–Impact of wider community**	Channelling communication through communities & religious groups	• Identify trusted local leadership and engage in reaching local communities • Utilise local community locations and hubs, including religious centres, through in-reach community vaccination programme delivery. • Consider the use of community ambassadors or champions to encourage vaccination uptake [32] • This report [33] makes further suggestions for community engagement
**Social Influence–Impact of closer group of family and friends**	Utilising inter-generational information transfer	• Ensuring vaccination promotion messages reach patients across age-groups and encourage sharing of information provision with family members across generations. • Use of social media and platforms commonly used by younger age groups to circulate vaccine information • Information-giving in a diversity of digital formats more likely to appeal to younger people
**Promotion of incentives for vaccination**	Highlight incentives for vaccination such as travel and protection of family members	• Explore potential vaccination benefits and their impact for the patient.

#### Concerns about the speed of vaccine roll-out

The speed of roll-out was widely cited, with some participants stating that as a result, they saw the program as experimental. More than half of participants across ethnicities raised specific concerns about the speed of vaccine design and implementation, including the majority of Black British participants, with associated suspicion that adequate testing had not been carried out. Several participants also questioned why scientists cannot find treatments and cures for conditions that have been around longer than COVID-19.

*“I think the wisest thing to do is just let things play out and take it last*. *I think everyone in my circle’s quite like that*, *just see what happens*.*”* P25, Black African, 31 years, Male

*“The cancer and AIDS*, *they’ve not been able to come up with anything substantial there for many*, *many years*, *but all of a sudden*, *for coronavirus*, *they’ve come up with some sort of vaccination that’s supposed to be 80% good*.*”* P7 Black Caribbean, 52 years, Male

More than one-third of participants expressed concerns and fears about the vaccination programme being experimental, with the term “guinea pigs” used specifically by four Black British participants. Several participants further expressed fears about racial experimentation and likened this to historical events.

*“And so it is an experiment*, *but it’s not acknowledging that there is an experiment*. *In fact*, *there are very few people who would say that we’re being experimented on*, *which in fact we are*. *In my opinion*.*”* P16, Mixed/multiple ethnicities, 60 years, Male

*“But now with this vaccine*, *everybody is going to be a guinea pig because they don’t know enough information”* P6, Black British Caribbean, 56 years, Female

*“I’m not sure because I think that they’ve been experimenting on Black people with the drugs all over the place*. *They would want for us to go and take this thing and if it works on us and they pass it on to everybody else*.*”* P15, Mixed White/Caribbean, 67 years, Male

#### Concerns about the vaccines’ known and unknown effects on health

A range of concerns relating to the vaccines were raised by participants, including the risk of side effects, the impact of vaccine ingredients, and uncertainty of the possible protection and benefits provided by the vaccine.

Concerns about side effects were mentioned by nearly all participants, particularly by participants with comorbidities. Concern over the harm of blood clots was the most frequently mentioned side-effect, with some participants also citing fear of the impact on fertility and pregnancy and even the risk of death. In addition, fear of unknown side effects that may present in the future was cited as a rationale to delay taking the vaccine, with the impact of the Thalidomide scandal cited several times.

“*I don’t want to put stuff in my body that’s going to make it worse… Why would I put Pfizer that in some studies*, *they’ve ended up with an enlarged heart and arrhythmia*?… *I already have those issues*. *I’m not going to exacerbate them*.” P29, Asian British, 56 years, Female

*“I worry about what I’ve heard*. *People are dying because they have clots”* P2, Black British, 68 years, Male

*“…At the time they were like*, *yes*, *it’s safe*, [Thalidomide], *it took them five years to realise the connection…So if here we are taking this vaccine during pregnancy … but at the same time*, *we don’t know the long-term effects*. *So why are you risking it*?*”* P38, British Asian, 31 years, Female

One-third of participants expressed uncertainty and concern about vaccine ingredients and the potential impact of these on health. Whilst this was in most cases not expressed as the main reason for vaccine delay, it was perceived as an additional contributor by several participants.

*“I just don’t trust all the chemicals that they (vaccines) put in your body*.*”* P11, Multiple/Mixed ethnicities, 56 years, Female

“*…The things that it has in it like element*, *like chimpanzees’ something… What else is it*? *I think it’s some kind of filling*…*”* P12, Black Caribbean, 56 years, Female

One-third of participants expressed concerns about whether having the vaccination would even protect them or others from coronavirus, a view expressed solely by Black British participants. This led them to question if the risks and uncertainty of the vaccination’s impact on their health were worth it.

*“Particularly me*, *I don’t know if you really are going to get protected if I got vaccination*?*”* P18, Black African, 40 years, Female

### Information sources of participants

Participants described a range of information sources to obtain an understanding of COVID-19 and COVID-19 vaccination. Television and radio programmes, internet searches and social media posts, including videos sent via WhatsApp, were commonly cited. Whilst, not all social media messages received from friends and family were believed, many participants demonstrated a high level of exposure to misinformation via this means. All participants gave examples of exposure to negative or concerning messages about vaccination. Several participants specifically addressed the idea of misinformation and had theories as to why inaccurate information was reaching them.

“*A lot of it* [information about coronavirus] *comes from WhatsApp*, *through the different groups that I’m part of*. *I do believe some of it is scaremongering*, *I’m not sure about the 5G…*.*”* P7, Black Caribbean, 52 years, Male

“*I think it was fear that was driving it (spreading misinformation) to be honest*. *It was such a difficult time and there were people who were really scared*, *there was almost like a paranoia*.*”* P20, Asian British, 40 years, Female

*“I guess because there was no information coming from the government*, *there was an empty space*, *and echo chamber*, *and people filled the space with conspiracy theories”* P10, Multiple/Mixed ethnicities, 50 years, Male

As well as discussing exposure to a range of information sources, a number of participants described a mistrust of information from official information sources, often resulting from mistrust of the authors specifically. One-third of participants expressed mistrust of government across a mix of ethnicities. One-fifth of Black British participants expressed mistrust in pharmaceutical companies, which in some cases were considered to be in league with the government. This mistrust contributed to mistrust in the vaccination, with participants expressing the view that these organisations had ulterior motives for wanting vaccination that did not account fully for participants’ safety. A number of participants were clearly not aware of official information on current available data on vaccination safety, and the research process that the vaccination had undergone.

*“And with the government rules*, *they’re breaking the rules*, *you know the stories that go around …how can you trust a government that keep on breaking the rules*?*”* P36, British Asian, 36yrs, Male *“Actually*, *the vaccines out there are making lots of money for pharmaceutical companies*. *And we know*, *I know*, *that there is actually far cheaper treatments out there that are equally as effective and actually I really don’t know how effective the vaccines are*.*”* P34, Black British Caribbean, 43 years, Male

Participants often described information originating from different sources, and in some cases, countries, as conflicting, and as a result they expressed uncertainty about what to believe, highlighting the ‘infodemic’ nature of this pandemic.

*“Basically*, *there’s a lot of conflicting information… if you listen to French news*, *you get some conflicting news… If you go the other side of Europe*, *similar… It has been conflicting*, *right across board*, *the whole world”* P26, Black British African, 70 years, Male

*“A lo*t [of information is available]. *Sometimes it’s only bad*, *but we don’t know what can we trust*? *It’s good*, *or it’s not good*. *Especially me*, *I end up in the middle*, *I don’t know*.*”* P18, Black-British, 40 years, Female

Interestingly, not all participants felt they wanted more information about the vaccination, as they already felt overwhelmed by the current information they had received. However, several participants stated that clearer messaging to develop a better understanding of the safety of the vaccine was needed and would help them or others they know to understand the vaccine better.

*“So short-term probably engagement in terms of leaflets and maybe little snippets of a video*, *say on the BBC*, *about the vaccine*, *and about its safety and something about clinical trials*. *Maybe some of this stuff is already out there*, *but if stuff like that goes viral it counteracts some of the negative and misinformed things that are in videos that are doing the rounds*.*”* P20, Asian, 40 years, Female

### Facilitators of vaccine uptake

There were several suggested mechanisms to improve vaccine uptake.

#### Trust in healthcare professionals

Participants were asked directly if they believed doctors were giving them correct information about COVID-19. Most participants expressed the view that they trusted information from healthcare professionals. When asked directly about their experience of healthcare and if they felt they were treated differently, all participants, except two, expressed the view that their experience of healthcare was good. Participants were then asked if there was anything GPs could do to change their minds about vaccine uptake. More than half of the participants agreed that an opportunity to receive clear and understandable information from their GP would be helpful to address vaccine hesitancy.

*“I feel like the doctors are in the same position as everyone else*, *trying to understand what’s actually going on*. *So*, *no*, *I don’t believe they’re giving incorrect information*.*”* P1, Black African, 30 years, Female

*“I think certainly the doctors*, *they can do a lot*. *Because the worry itself can kill*, *you know*. *But if a person speaks to a doctor and the doctor answers calmly and tries to explain”* P33, Black British, 61 years, Male

### Utilising communities and social influence

#### We asked participants about their views on the role of celebrities, community leaders, and religious leaders in promoting vaccination

Most participants expressed that they did not feel seeing celebrities taking the vaccine would influence them. The justification often given was that celebrities were not seen to be any different from them, being described as “normal people” or “human beings” and did not appear to be a particularly trusted source.

*“Celebrities are just celebrities*, *they’re just ordinary people just like you and I*. *Them taking the vaccine doesn’t mean anything and I can’t prove they’ve actually taken the vaccine*.*”* P5, Black Caribbean, 82 years, Female

There was variability amongst participants about the role of community leaders which appeared to be linked to how well-embedded the individual was in their community. More than half of participants who appeared to have strong community links or knowledge of local leaders felt they would have a role in vaccination promotion. A few participants recognised religious leaders as having a role in vaccination promotion for those with religious beliefs and for those who attended religious institutions regularly

*“I think it’s about [community leaders] taking it themselves*. *By demonstrating that they’ve had taken it and over time showing them that everything’s okay*.*”* P12, Black Caribbean, 56 years, Female.

*“In the ethnic minority community*, *churches would be a good way*. *So through the pastors*, *that would be*, *or if it’s Islam*, *it should be the imam*, *that would be very good*.*”* P10, Mixed African/Caribbean, 50 years, Male

#### Promotion of incentives for vaccination

When considering facilitators that would encourage them to get the vaccine, some participants mentioned external incentives as motivators; these included being able to travel and see older family members. In addition, one participant suggested how vaccination could impact everyday freedoms, such as the ability to attend sporting events safely.

*“Get the vaccine now*, *and in three months’ time I will be flying out of the country for a little while*, *I do that every year*. *And they said we have to have two*. *So I want this year to get the other one*, *that’s the reason*…*”* P5, Black Caribbean, 82 years, Female

*“I think what was in the forefront of my mind is my elderly mother*. *So I thought that that was really important for the family time*, *but then also*, *I wanted to get on with my life*.*”* P24, Black African, 45 years, Female

*“Imagine a football stadium at the turnstiles…To watch our matches you’ve got to have a vaccination*. *So it’s really those practical real life situations that will get people’s attention*.*”* P22, Black African, 58 years, Male

## Discussion

### Summary

In this study of the barriers and facilitators of vaccine uptake in ethnic minority groups, participants highlighted a diversity of reasons for COVID-19 vaccine hesitancy which were grouped into the following key themes: concerns about the speed of vaccine rollout, concerns about the vaccines’ known and unknown effects on health, mistrust of official information and exposure to misinformation. Many participants demonstrated high exposure to misinformation from social media, family and friends. This led to uncertainty and a sense of conflicting messages surrounding vaccination. In addition, some participants were mistrustful of government and pharmaceutical companies and their motives for vaccine deployment, which led to mistrust of official information sources.

Participants identified that positive vaccine messages from trusted professionals such as GPs would help change their minds about vaccination. Some respondents stated that incentives, such as the ability to travel and see older family members, increased their likelihood to have the vaccine. Key implementation messages addressing the barriers and facilitators identified by participants have been highlighted in **Table 2**.

### Strengths and limitations

This is the first exploratory study, to our knowledge, in UK primary care that directly examines the views of COVID-19 vaccination from ethnic minority groups which have previously been identified as having the lowest COVID-19 vaccine uptake and highest reported vaccine hesitancy. Patients were diverse in terms of age, gender and educational backgrounds and reported a wide range of views and beliefs. The validity of the study is enhanced by using iterative data collection and analysis concurrently and in-depth interviews to gain a richer understanding. The findings have direct implications and applicability for the current COVID-19 vaccination programme. Of note, during study recruitment and analysis, the vaccine schedule was updated with more participants becoming eligible.

Although all the researchers agreed on reaching data saturation, the numbers of some ethnic groups in the study are relatively small, and the groups themselves are diverse with a wide range of social, cultural and historical differences. Beyond this exploratory study, further research could seek to find out the variations between different ethnic groups and age-related differences in more detail. The research team attempted to consider how their backgrounds influenced the interpretation of the data through active reflexivity; however the team’s ethnicities did not match the ethnic backgrounds of the participants. Therefore, some participants’ responses may have been influenced by the researchers’ professional training [34], relationship to the clinical practice, social backgrounds, and ethnicity. Further, the views expressed may have been affected by social desirability bias. Some vaccine-hesitant individuals were reluctant to speak freely about their views, and under half of unvaccinated people, particularly people of Black ethnicities, declined the invitation to participate in the study. Although we tried to include patients with limited English proficiency by offering interpreters and translated information leaflets and consent forms, it is possible that they were excluded due to language barriers. Therefore, selection bias could limit findings. Participants were interviewed at one time point; therefore, we do not know how participants’ decision-making regarding vaccination progressed.

### Comparison with existing literature

There is emerging qualitative and quantitative evidence on possible reasons for COVID-19 vaccine hesitancy, which aligns with our findings [1,35,36]. However, to date, none of the studies has specifically focused on a range of views from different ethnic minorities with the highest vaccine hesitancy; instead, previous similar work has focused on smaller subsets [36]. Further, given that two-thirds of phase 1 COVID-19 vaccinations were deployed by primary care in the UK [37], our findings provide insights into specific facilitators that could be utilised in this setting.

The majority of participants did not have qualifications beyond A-levels; this resonates with a previous study on global trends in vaccine confidence, which identified that fewer years of education was associated with decreased vaccination uptake [38]. Previous studies have demonstrated that a belief in conspiracies is the biggest predictor of vaccine hesitancy [39], several of our participants shared views aligning with conspiracy theories. All demonstrated exposure to vaccine-negative messaging and unsubstantiated theories about the vaccine. The widespread, pervasive nature of misinformation during the pandemic has led to the recognition of an ‘infodemic’, undermining vaccination efforts [40,41]. Once a negative heuristic bias develops, studies have shown that all subsequent information exposure, either sought or provided through social media, reinforces negative perceptions [42,43]. Social norms sustain this via shared perspectives and information from friends and family.

Attitudes surrounding vaccination from specific groups may ultimately reflect a more general underlying world view or “attitude root”, which manifests to motivate rejection of scientific evidence [42]. This study points to some of the possible underlying fears, ideologies, and experiences of certain ethnic groups linked to societal historical racism and inequity, which may drive cognitive biases surrounding vaccination decision-making. The recent evidence from the UK supports this narrative.

The findings also concur with previous recommendations on the need for targeted information, resources and support services around the COVID-19 vaccine for individuals from ethnically diverse backgrounds [44], and the need to partner directly with the local community and faith groups to co-design solutions for vaccine uptake for vulnerable groups [45,46].

The endorsement from trusted healthcare professionals has been one of the most powerful correlates of vaccine acceptability among patients [47], this relies on a patient trusting the healthcare professional greater than other sources of information for their health (e.g. friends and social media) [38]. Trust is an intrinsic and potentially buildable factor in vaccine acceptance, and previous pandemics such as Ebola, MERS and SARS have demonstrated the value of trusted guidance and information sources [48] in the management of the pandemics. The findings of this study suggest opportunities for patients to have conversations with healthcare professionals, such as GPs, may help overcome vaccine hesitancy. This may reflect the trust patients have in their own GPs.

### Implications for practice and research

This study provides valuable new insights into the barriers and facilitators of vaccine uptake in ethnic minorities in the UK. Given the high mortality of COVID-19 amongst these groups, the results are immediately relevant to the current COVID-19 vaccine deployment and efforts to encourage ‘booster’ vaccination.

#### Interventions and recommendations

Interventions for practice include tailored public health, in-reach campaigns to develop trusted relationships between local communities and healthcare providers delivering vaccination, working to address the concerns of these groups specifically. We know that knowledge provision alone does not lead to active behaviour change [49], therefore the importance of partnership with local community and faith groups is critical, alongside leveraging the trusted relationship between healthcare professionals and patients to promote vaccination uptake. Supportive environments and tailored messaging within communities are crucial for making positive behaviour change possible as underscored by the Behaviour Change Communication principles [50,51]. The Capability-Opportunity-Motivation-Behaviour (COM-B) and Theoretical Domains Framework (TDF) are commonly used to guide work on barriers and facilitators to behaviour change [52,53]. The TDF defines 14 domains of influence on behaviour change, including factors such as social influences (e.g., social persuasion, support and comparison) as well as social identity, knowledge, emotion and reinforcement (e.g., social gains and sanctions for being vaccinated or not). Combatting widespread misinformation shared via social media and through informal networks of family and friends is challenging [40]. Provision of clear, consistent, positive vaccine messaging by trusted local primary care professionals and community leaders (via electronic or other format) alongside an opportunity for further discussion (either through group meetings or at the vaccine clinic) is one step to active participation. Encouraging patients from ethnic minorities who have chosen to be vaccinated, to share positive vaccination stories, and beliefs, with family and friends, could be used to harness the potential of social influence and intergenerational information transfer. Table 2 provides a number of considerations and resources for practice.

Future research should explore the similarities and diversities between our findings and the facilitators and barriers to uptake in other vaccine-hesitant population groups such as those from lower socioeconomic backgrounds, Gypsy, Roma and Traveller populations and migrant groups amongst others, to consider generalisability [54]. Projects aiming to improve vaccine uptake in these groups require rigorous evaluation to determine effectiveness and scalability [55].

Many individuals from ethnic minorities remain unvaccinated across the UK, and in the context of high COVID-19 infection rates, future policy efforts and funding must focus on mitigating those risks through targeted campaigns and support to prevent widening health inequalities and inequities.

### Conclusions

The COVID-19 pandemic has led to disproportionately high cases, hospitalisation and mortality in ethnic minority groups, who already face major disparities in health outcomes [18]. In addition, evidence clearly shows a higher vaccine hesitancy among some ethnic minority groups and consequently lower COVID-19 vaccine uptake.

This is the first exploratory qualitative study about the specific barriers and facilitators in ethnic groups identified as having the lowest COVID-19 vaccine uptake and highest reported vaccine hesitancy. This primary-care based study can help tailor public health campaigns to specifically address the concerns of these groups regarding experimentation and vaccine ingredients, as well as providing reassurance on the speed of vaccine-roll out and the known side effect profile. Vaccine providers can equip themselves to support individuals from these ethnicities to make informed decisions about whether to take a COVID-19 vaccine by seeking to understand previous misinformation exposure, providing tailored information, addressing how different ethnicities were involved in vaccine studies to date and harnessing social influence and intergenerational information transfer through partnering closely with local community and faith groups.
